# U-Index, a dataset and an impact metric for informatics tools and databases

**DOI:** 10.1038/sdata.2018.43

**Published:** 2018-03-20

**Authors:** Alison Callahan, Rainer Winnenburg, Nigam H Shah

**Affiliations:** 1 Stanford Center for Biomedical Informatics Research, Stanford University, Medical School Office Building X215, Stanford, CA 94305, USA

**Keywords:** Publishing, Research data, Literature mining, Databases, Software

## Abstract

Measuring the usage of informatics resources such as software tools and databases is essential to quantifying their impact, value and return on investment. We have developed a publicly available dataset of informatics resource publications and their citation network, along with an associated metric (u-Index) to measure informatics resources’ impact over time. Our dataset differentiates the context in which citations occur to distinguish between ‘awareness’ and ‘usage’, and uses a citing universe of open access publications to derive citation counts for quantifying impact. Resources with a high ratio of usage citations to awareness citations are likely to be widely used by others and have a high u-Index score. We have pre-calculated the u-Index for nearly 100,000 informatics resources. We demonstrate how the u-Index can be used to track informatics resource impact over time. The method of calculating the u-Index metric, the pre-computed u-Index values, and the dataset we compiled to calculate the u-Index are publicly available.

## Introduction

With advances in information technology and methods for data analysis, significant resources have been devoted to developing informatics software tools and databases to support biomedical research. Quantifying the usage of such resources is essential for measuring their value and return on investment; however, information about dissemination and use is difficult to obtain. Previous work to track resource usage has involved developing registries and repositories^1–7^, or “resourceomes”^8^, as well as methods to populate them^9–11^ including methods to detect mentions of resources in research publications^12–14^ as a proxy for usage. Registries require significant curation effort to maintain and can become out-of-date, while automated methods for detecting resource names and URLs in text may be inaccurate. There are additional challenges in measuring usage by secondary analysis of publication text and citations. For example, not all citations of a resource in the body of a research article are a signal of usage.

Despite these challenges, citation data have been extensively used to measure author impact^15^, quantify article similarity^16^, and analyze the structure of academic fields^17^. For researchers developing informatics tools and databases, peer-reviewed publication remains the primary means of disseminating their work and thus citations to these publications are an important mechanism for measuring impact. Entire journals and article types are dedicated to publishing descriptions of tools and databases in specific domains, such as the annual Nucleic Acids Research Database and Web Server issues, Oxford Database, Nature Scientific Data, and Bioinformatics Application Notes. The National Library of Medicine also maintains vocabularies and comprehensively indexes published biomedical literature, which allow us to search for and organize literature by subject. These journals and article databases comprise a valuable resource for quantifying impact using citation data, but one which requires significant processing to extract this value.

Therefore, we have curated publicly available literature citation records to create a dataset of informatics resource publications, their citations, and citation location (to distinguish usage versus awareness), as well as associated methods for measuring usage and impact. To demonstrate one way our curated citation dataset may be used, we developed the *u-Index*, a measure of impact for informatics tools and databases that distinguishes between usage and awareness of a tool or database using citation data. The u-index is similar in spirit to the h-Index^18^, which measures the impact of individual researchers based on their number of publications and the citations of those publications. We make the dataset, PubMed queries, and code for processing article records to quantify usage as well as calculate a resource’s u-Index publicly available so that they can be used and updated over time. An advantage of measuring resource impact via a publicly available, and updateable dataset is the resulting transparency of the approach—although no method that relies on these sources can avoid the issue of potential missingness. An additional benefit of our approach is that it discerns resource use from other types of citation by considering the location of citations within publications.

## Results

### Citing universe

To quantify how informatics resource publications are cited, we first processed the PubMed Central (PMC) Open Access subset (Table 1) to build a ‘citing universe’, including the article section(s) in which citations occur. We define a citation as a structured reference to a publication in the body of an article, which has a corresponding listing in the References section of that article where information about the publication including authors, title and source journal, book or conference are detailed. We refer to an article containing a citation as a ‘citing article’. Our citing universe consists of a large set of such citing articles. We distinguish between two types of citation: a ‘usage’ citation is one that occurs in the Materials/Methods section of an article while an ‘awareness’ citation is a citation that occurs in any other section of an article. To ensure a fair comparison, we restricted our citing universe from 1,152,905 PMC articles to the 691,527 research articles that have a Materials/Methods section. Of these, 669,714 (90%) have at least one literature citation (of which 661,782 had at least one literature reference with a PubMed identifier provided). 596,627 (71%) articles have a Materials/Methods section that contained literature citations (of which 543,661 had at least one literature reference with a PubMed identifier provided).

### Informatics tool and database publications

We queried PubMed to retrieve 101,001 research publications describing informatics tools and databases (see Methods section for query details; we will refer to this collection of publications as ‘informatics resources’ throughout this article). For informatics resource publications in Bioinformatics Applications Notes, the Nucleic Acids Research Database special issues, and Oxford Database (Table 2), our query recall was 91%. For the 250 articles with the most usage citations in the citing universe, query precision was 97% and recall was 77% (Table 3).

We processed the set of informatics resource publications to automatically aggregate publications that are about the same tool or database to a single resource ‘key’ (for example, there are 8 publications about the Gene Ontology^19^ in the set of informatics resource publications; see Methods), and verified the correctness of this aggregation for articles with 50 or more citations. The set of 101,001 publications corresponds to 99,992 unique resources.

To empirically quantify the types of citation to these resources that occur in the Materials/Methods sections of articles, we randomly selected 160 citations identified by our method as usage citations (10 from each of the publication years included in our study, 2000-2015) and manually assessed each one to determine if it was indeed a usage citation. Of the 160 citations, 139 (86.9%) were direct usage citations. The remaining 21 citations were categorized as follows: 10 referenced results from the original resource publication, which is itself a kind of usage; 6 were citations that compare the citing work to the cited resource; 2 referenced resources that the citing publication is based upon; 1 referenced a resource supported by the citing work; 1 referenced a publication written by a 3rd party describing a resource; and 1 was due to an error in the citing article’s PMC XML. None of the citations had a negative context.

### Informatics resource use in different domains

**Figure 1:**
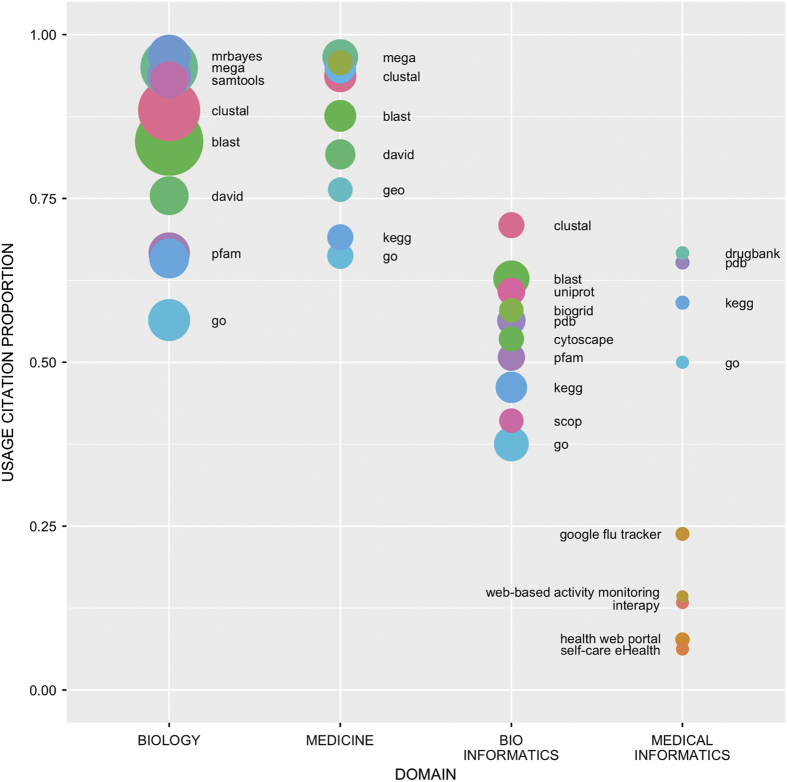
The proportion of usage citations for informatics resources across the domains of biology, medicine, bioinformatics and medical informatics. The X-axis shows the four domains and the Y-axis shows the proportion of papers in which the tool is used in comparison to the total number of citations of this tool. The size of the bubble is proportional to the total citations for each tool. Each color is tool specific and conserved across the four domains, e.g. BLAST is consistently shown in green.

We quantified how informatics tools and databases are used in the domains of biology, medicine, bioinformatics and medical informatics by restricting the citing universe (Fig. 1; see Methods for details on how we restricted the citing universe by domain). Papers describing resources such as the Gene Ontology^19^ and tools such as BLAST^20^ and CLUSTAL^21^ have more usage citations in biology and medicine than in bioinformatics and medical informatics. In informatics domains, these resources have high citation counts, but they are cited in an awareness context more often than in a usage context. This is consistent with the goals of research in informatics where researchers who develop informatics tools compare and discuss related tools; medicine and biology research increasingly *use* informatics tools in data processing and analysis.

### The u-Index metric

Inspired by author-level impact metrics such as the h-Index and the observed differences in resource usage across domains, we developed the u-Index – a resource-level measure based on the number of resource usage citations, the ratio of usage citations to awareness citations and the age of the resource (in years since first publication) to quantify resource impact. The formula for calculating the u-Index is:

$$u-\mathrm{Index}=\frac{\mathrm{total}\#\mathrm{citations}*\mathrm{usage}\mathrm{ratio}}{\#\mathrm{years}\mathrm{since}\mathrm{first}\mathrm{publication}}$$

The ‘total # citations’ is the number of citations of any kind that a resource has in the citing universe. The usage ratio is the ratio of the number of usage citations to the number of awareness citations for a given resource:

$$\mathrm{usage}\mathrm{ratio}=\mathrm{usage}\mathrm{citations}:\mathrm{awareness}\mathrm{citations}=\frac{\#\mathrm{usage}\mathrm{citations}}{\#\mathrm{awareness}\mathrm{citations}}$$

Resources with a high number of citations, a usage ratio greater than 1, and which have been recently published, will have a high u-Index. Resources with a very high number of citations and a modest usage ratio will ‘beat out’ resources with a very low number of citations but a usage ratio close to or greater than 1. This captures the notion that a very highly cited resource is clearly impactful, even if these citations are not always in a usage context. The above formula can be used to calculate the u-Index for a resource using all available data, data up to a specific year, or even a single year of citation data. We include the ‘number of years since first publication’ to normalize for the effect of resource age on number of citations - informatics resource publications that have been available for many years are more likely to have higher citation counts.

### Data and code availability

We have calculated the u-Index for all resources and articles in the set of informatics resource publications returned by our PubMed query, based on citations in the citing universe of PubMed Central articles with a Materials/Methods section. We have constructed a u-Index dataset consisting of the aggregated resource key or PubMed identifier for each informatics resource, the citation counts, usage ratio and u-Index from 2000 to 2015 (the most recent year for which PubMed and PubMed Central records were available at the time of data analysis). This dataset is publicly available (Data Citation 1). The code to generate this dataset from PubMed and PubMed Central records is available at https://github.com/alisoncallahan/uIndex_data_generator.

## Discussion

**Figure 2:**
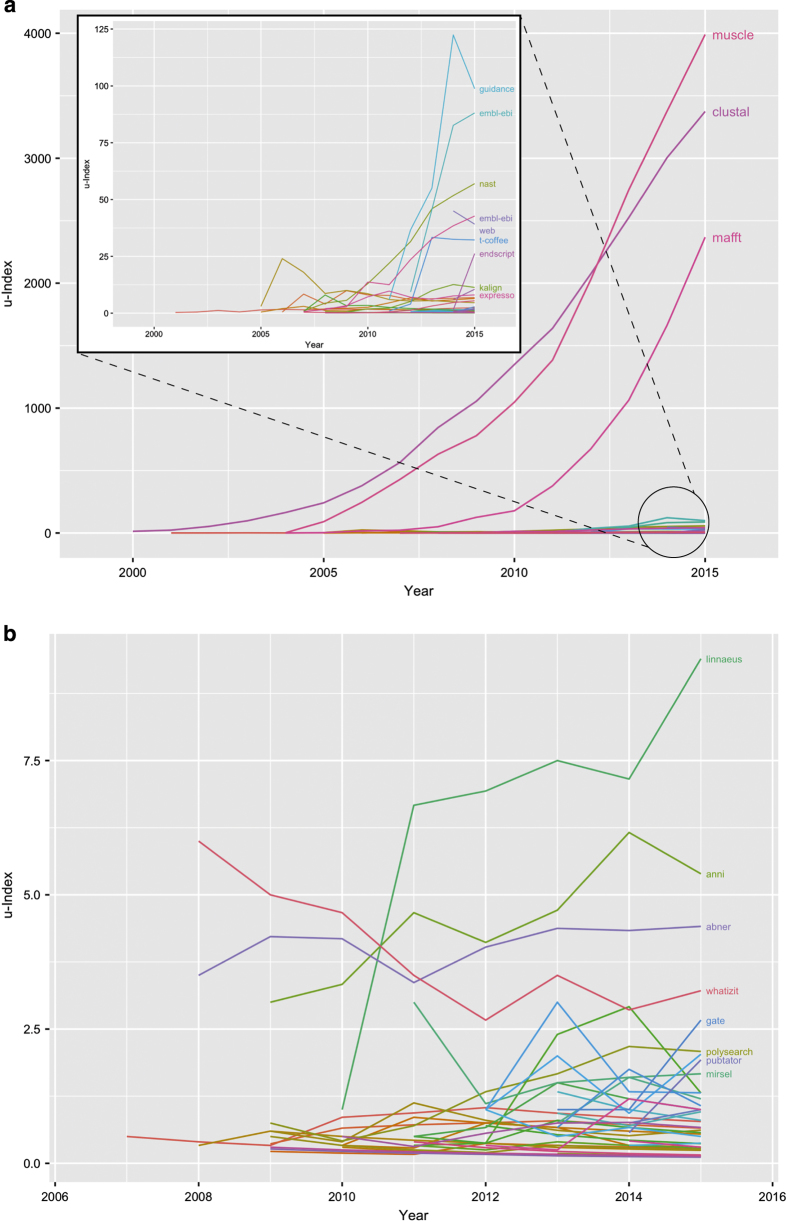
u-Index values over time for multiple sequence alignment tools and text mining tools. Multiple sequence alignment tool u-Index values are shown in panel. (**a**), and text mining tool u-Index values are shown in panel (**b**). The X-axis shows the years a tool has been cited and used and the Y-axis shows the u-Index for a tool, based on the cumulative usage and awareness citations up to and including that year. Three tools dominate among the multiple sequence alignment tools – Muscle, Clustal and MAFFT. Text mining tools, on the other hand, have lower u-Index values, with the most reused tool being LINNAEUS, used to identify species names in text.

There are many potential uses for our dataset and the derived metric of informatics resource impact. Developers of informatics tools or databases can measure the impact of their tool/database compared to other resources of a similar age or in the same category. For example, Fig. 2 shows the u-Index over time for multiple sequence alignment (MSA) tools published in Nucleic Acids Research (Fig. 2a) and for text mining tools (Fig. 2b). The publications describing MSA and text mining tools included in Fig. 2 were selected via PubMed queries (see Methods). MSA tools vary widely in their u-Index values, with 3 tools dominating -  Muscle^22^, Clustal^23^ and MAFFT^24^. Our method successfully aggregated the articles published on each of these resources to pool their citation counts when calculating the u-Index. Text mining tools, on the other hand, have much lower u-Index values overall, with LINNAEUS^25^, a tool for identifying species names in text, having the highest u-Index of the set (Fig. 2b). In comparison to MSA tools, text mining tools are relatively recent entrants to the informatics resource universe. These examples demonstrate how the u-Index can give insight into the relative usage of informatics resources, but other applications are possible as well. For example, for researchers, the u-Index can help to answer the question “what tool/database(s) should I use?”. Tools or databases with a higher u-Index, and are more used, have a larger community that may be able to provide support, as well as indicate the degree of success users have in using the resource.

A key simplifying assumption of our methods is that traditional citations are reflective of how a resource is perceived and used within the scientific community. This assumption has its pitfalls^26,27^, including that citation practices are often biased towards well-known and previously well-cited publications, and, as previously described, not all resources are cited when used. Our proposed approach to analyze the dataset of informatics resource publications and citations is only one of many approaches for measuring impact. By making the dataset we use publicly available, along with the analysis code, we invite others to devise novel analysis regimes for these data. Significant decisions rely on such measures of research impact, from students choosing a research project to allocation of national funds. We hope that this dataset will be combined with additional data and methods, to develop increasingly accurate measures of impact.

Additional limitations to the dataset and index we have developed result from the approach used to identify literature describing informatics resources and the potential ambiguity of the citing-cited relationship. While our methods for identifying informatics resource literature are accurate, as evidenced by the precision and recall metrics in Table 2 and Table 3, our PubMed query to retrieve articles that describe informatics resources missed some relevant articles and also retrieved articles that do not describe an informatics resource. The universe of articles for which we have calculated a u-Index value is therefore not complete. As a result, resources that are described in multiple publications, and for which a relevant publication is not retrieved by our query, might have a lower u-Index value.

The u-Index is naturally sensitive to the choice of citing universe used to derive usage and awareness citations, as is any method that relies on citations to derive a metric of usage or impact. In this work, we used research articles from the PubMed Central (PMC) Open Access Subset. Our motivation for constructing our citing universe from the PMC Open Access Subset is two-fold: firstly, it allows anyone to reproduce our work because it does not rely on institution specific access to bibliographic databases. Secondly, the structured nature of the PMC Open Access subset makes it possible to extend our work in calculating the u-Index, for example by restricting the citing universe to exclude self-citations or citations within an author’s co-author network. A strength of the methods we propose and the code we developed that implements them is that they do not rely on a specific citing universe or set of cited articles. Anyone may define their own citing universe and measure the u-Index for a given set of articles based on this universe.

With regard to ambiguity in the citing-cited relationship as a reflection of usage, it is possible that an informatics resource is used but not cited in the Methods section of an article, or is not cited at all. Any approach that analyzes publications to measure resource usage faces the challenge of under-reporting due to missing resource attribution as mentions or structured citations^28,29^. Ours is not the first method to distinguish between resource usage and resource awareness using information about article structure – Duck et al.^12–14^ also use article section information to measure tool and database use. Limitations to their approach stem from inaccuracies in detecting resource name mentions in unstructured text. For example, such methods cannot consistently disambiguate mentions of ‘muscle’ the tool name from ‘muscle’ the tissue. Alternative approaches for quantifying the impact of a resource, such as Altmetric^30^, address this by including citations and mentions from social media sites, news articles, and blogs. It would be challenging to analyze such additional sources because they lack the structure of a scientific publication to distinguish ‘usage’ citations from ‘awareness’ citations. Finally, it is possible, although unlikely, that the citation of an informatics resource is due to its disutility. We do not analyze the context in which citations occur beyond determining the article section in which a resource was cited. As a result, citations with a positive or negative sentiment are not distinguished. However, negative citations were not observed in our analysis of a random subset of resource usage citations, which suggests that they are a rare occurrence.

In summary, we have developed a publicly available dataset of informatics resource publications, and their citation network, along with an associated metric to measure informatics resources’ impact over time. The dataset and u-Index values, as well as the queries and code to generate them are publicly available so that they can be refreshed over time.

## Methods

### Defining a citing universe from PubMed Central

To construct the citing universe (articles which potentially cite informatics resource articles), we downloaded the XML records of journal articles in the open source subset of PubMed Central® (PMC)^31^, the full-text archive of biomedical and life sciences journal literature at the U.S. National Institutes of Health National Library of Medicine (NIH/NLM). We restricted the universe to research articles using the headings assigned to articles by PMC, by removing articles with the headings listed in Table 4. We further filtered this set to articles with a Materials/Methods section, which we identified by the section type attribute or - if missing - the section title.

References from the reference list section (denoted by the ‘<ref-list>’ tag) at the end of each article were associated to citations in the body of the article using cross-references (denoted by the ‘<xref>’ tag) in the text body, to determine in which sections they were cited. We also resolved references that are not explicitly mentioned in the text but implicitly in reference enumerations, such as “[2-5]”, where possible.

### Querying PubMed for informatics resource articles

We designed a PubMed^32^ query that uses MeSH^33^ index terms to retrieve articles describing the development or extension of a database or tool whose intended use is in the biomedical domain from MEDLINE^34^. We filtered by article type to exclude reviews and newspaper articles. For the resulting PubMed identifiers, we retrieved the XML files of their publication record from MEDLINE using NCBI Entrez Programming Utilities (E-utilities)^35^, from which we extract titles, abstracts, author names, publication dates, and MeSH term annotations for each article.

The PubMed query for retrieving informatics resource articles is:

("software"[MeSH Major Topic] OR "databases, factual"[MeSH Major Topic:noexp] OR "databases, protein"[MeSH Major Topic:noexp] OR "databases, nucleic acid"[MeSH Major Topic:noexp] OR "databases, pharmaceutical"[MeSH Major Topic:noexp] OR "databases, chemical"[MeSH Major Topic:noexp] OR "databases, genetic"[MeSH Major Topic:noexp]) OR ("software"[MeSH Terms] AND ("databases, factual"[MeSH Terms:noexp] OR "databases, protein"[MeSH Terms:noexp] OR "databases, nucleic acid"[MeSH Terms:noexp] OR "databases, pharmaceutical"[MeSH Terms:noexp] OR "databases, chemical"[MeSH Terms:noexp] OR "databases, genetic"[MeSH Terms:noexp])) OR ("algorithms"[MeSH Terms] AND ("databases, factual"[MeSH Terms:noexp] OR "databases, protein"[MeSH Terms:noexp] OR "databases, nucleic acid"[MeSH Terms:noexp] OR "databases, pharmaceutical"[MeSH Terms:noexp] OR "databases, chemical"[MeSH Terms:noexp] OR "databases, genetic"[MeSH Terms:noexp])) OR ("algorithms"[MeSH Terms] AND "software"[MeSH Terms]) OR ("computer communication networks"[MeSH Terms] AND ("databases, factual"[MeSH Terms:noexp] OR "databases, protein"[MeSH Terms:noexp] OR "databases, nucleic acid"[MeSH Terms:noexp] OR "databases, pharmaceutical"[MeSH Terms:noexp] OR "databases, chemical"[MeSH Terms:noexp] OR "databases, genetic"[MeSH Terms:noexp]))

Similarly, the resources whose u-Index values are shown in Fig. 2 were selected by intersecting the informatics resource set with the results of the following PubMed queries:

Multiple sequence alignment tools: "multiple sequence alignment" AND SOFTWARE[MAJOR] NOT Review[Publication Type] AND "Nucleic acids research"[Journal]

Text mining tools: "text mining" AND SOFTWARE[MAJOR] NOT Review[Publication Type]

### Aggregating articles about informatics resources

To aggregate articles describing the same resource, we used a rule-based approach to extract resource names from article titles and abstracts. For example, if an article title consists of a series of words followed by a colon, our system extracts the words preceding the colon as a resource name. If a title contains a word that is entirely upper case, the word is extracted as a resource name. Lastly, if a title contains a version number, we extract the words up to and including the version number as a resource name. We then apply a normalization step, in which we remove capitalization, hyphenation, or version information from the extracted resource name. For example, *T-Coffee* is converted to *tcoffee*, *MrBayes 3.2* to *mrbayes*, etc. If a resource name cannot be identified, the resource key is its PubMed identifier. We manually reviewed resource keys for the articles with 50 or more citations in the citing universe, and the 5,458 articles published in our gold standard.

Using the normalized resource name keys, we collected the PubMed identifiers of all articles written about the same resource. For example, there are more than 10 papers on the resource Pfam^36^. The u-Index for a resource is calculated using citations of all articles written about that resource.

### Counting usage and awareness citations

When processing the citing universe to count usage and awareness citations for an informatics resource, each citing article contributed at most 1 to the tallies of ‘usage’ and ‘awareness’ citations. That is, if an informatics resource was cited multiple times within a single article, it was counted as a single citation. If there was at least one citation to an informatics resource within an article, we incremented the *total citation count* for that resource by 1. If there was at least one citation to an informatics resource within the Materials and Methods section of an article, we incremented the *usage citation count* for that resource by 1. The *awareness citation count* for an informatics resource was calculated as the difference between the *total citation count* and the *usage citation count*. For example, if an informatics resource had a *total citation count* of 10 and a *usage citation count* of 2, its *awareness citation count* was calculated as 10 - 2=8. The usage ratio for that resource was then calculated as 2:8, or 0.25. In the case that total citation count equaled the usage citation count and the resulting usage ratio was mathematically undefined, we set the usage ratio as 1, making the u-Index equal to the total number of citations. This was the case for ~5% of informatics resources. We chose this method because we consider usage as an additive factor in determining resource impact alongside, but not exclusive of, total citation count.

### Categorizing biomedical research articles into subdomains

We assigned biomedical research articles from our citing universe to one or more of the four subcategories: Biology, Medicine, Bioinformatics, and Medical Informatics. This assignment is based on the journal in which the article was published, using the MeSH Broad Subject Terms for indexed journals in the NLM catalog. Journals were automatically categorized if they were tagged with any of the four head terms ‘Medicine’, ‘Biology’, ‘Computational Biology’, or ‘Medical Informatics’, or any of their child terms. Journals that are tagged with subject terms that are relevant but without hierarchical relations to any of the four head terms, e.g. *Therapeutics*, were manually assigned any of the four categories. Articles from journals with a broad scope, i.e. having more than two head terms, such as *PLoS ONE*, were not assigned to any subcategory. Also, journals that are not currently indexed by the NLM (e.g., *Frontiers in Psychology*) could not be assigned to any subcategory. Of note, articles that could not be assigned to any category are excluded from our domain specific analysis (Fig. 1) but were included in the citing universe and used when calculating u-Index values. Table 5 shows the number of articles assigned to the four categories in our citing universe.

### Evaluating PubMed query performance

We approximated query recall by measuring how many articles from three relevant journals are captured in the informatics resource set retrieved by our PubMed query:

- Nucleic Acid Research Database Special Issues from 2004-2015
- Oxford Database from 2010-2015
- Application notes in the journal Bioinformatics from 2005-2015

We also approximated query precision, recall and specificity by selecting the 250 articles with the most usage citations in our informatics resource article set and manually assessing whether each article is an article about an informatics resource. All articles were assessed independently by authors RW and AC. Results were compiled and disagreeing assessments were resolved in a joint effort. We used information from the abstract and full text (where available) to address the following criteria:

- Was the resource made available at the time the article was published (e.g. as a database, software, website, etc.)?
- Does the article describe an informatics resource (e.g., excluding lab protocols, hardware drivers)?
- Is the resource the primary topic of article (e.g., excluding secondary usage publications)?

We determined how many of the articles are true informatics resource articles using the criteria above and how many are captured in the set of informatics resource articles retrieved by our PubMed query.

### Creating the u-Index dataset

The Python code we developed to generate the u-Index dataset from PubMed and PubMed Central records is available at https://github.com/alisoncallahan/uIndex_data_generator. The code consists of scripts to (i) extract citations from PMC XML files (‘PMCReferenceExtractor.py’), (ii) query PubMed for informatics resource articles (‘PubMedPapersQuery.py’), (iii) extract information from the retrieved records (‘PubMedPapersProcessor.py’), (iv) extract resource names from these records (‘ResourceNameExtractor.py’) and (v) load the output of each of these scripts into a SQL database (‘DBLoader.py’). We also provide a script to run each of these processes in sequence (‘runner.py’).

## Additional information

**How to cite this article**: Callahan, A. *et al.* u-Index, a dataset and an impact metric for informatics tools and databases. *Sci. Data* 5:180043 doi: 10.1038/sdata.2018.43 (2018).

**Publisher’s note:** Springer Nature remains neutral with regard to jurisdictional claims in published maps and institutional affiliations.

## Figures and Tables

**Table 1:** Details of the PubMed Central Open Access articles used as a citing universe.

	Articles (n = 1,152,905)	Research		Other	
Articles (n = 1,152,905)	691,527		461,378	
Articles with references	669,714 (97%)		329,969 (72%)	
	All	With PMIDs	All	With PMIDs
# References	27,218,513	21,130,489	12,594,333	9,563,297
Average # references				
All articles	39.4	30.6	27.3	20.7
Articles with references	40.6	31.6	38.2	29.0
Articles with references in methods	596,627 (89%)		18,401 (6%)	
	All	With PMIDs	All	With PMIDs
# References in methods	4,734,310	3,230,941	193,310	138,650
Average # references				
All articles	6.8	4.7	0.4	0.3
Articles with refs. in methods	7.9	5.4	10.5	7.5

**Table 2:** Recall of the PubMed query for informatics resource articles published in Bioinformatics Application Notes, the NAR Database special issue, and Oxford Database.

**Article collection**	**# Articles**	**# In informatics resource publications**	**Recall**
Bioinformatics Application Notes	2,913	2,540	0.87
NAR Database	2,029	2,024	0.99
Oxford Database	516	408	0.79
Total	5,458	4,972	0.91

**Table 3:** Precision, recall, and specificity of the PubMed query for informatics resource articles, for the 250 articles with the most usage citations in the PubMed Central Open Access subset.

**Article collection**	**TP**	**FP**	**TN**	**FN**	**Precision**	**Recall**	**Specificity**
250 articles with the most usage citations in PMC	116	3	97	34	0.97	0.77	0.97

**Table 4:** Headings used to filter the PubMed query to retrieve informatics resource articles.

addendum/addenda	erratum/errata
brief communication	interview
clinical overview	opinion
column	perspective
comment	reply
correction	report
editorial/editor	review

**Table 5:** Number of articles and journals assigned to the four categories in the citing universe.

**Domain**	**Number of articles**	**Number of journals**
Medicine	253,729	1,934
Biology	157,567	1,021
Bioinformatics	12,289	18
Medical Informatics	3,151	23
Total	691,527	2,548
